# Trends in hospitalization and in-hospital mortality rates among patients with lung cancer in Spain between 2010 and 2020

**DOI:** 10.1186/s12885-022-10205-2

**Published:** 2022-11-21

**Authors:** Abraham Galindo-Utrero, Jesús María San-Román-Montero, Ruth Gil-Prieto, Ángel Gil-de-Miguel

**Affiliations:** 1 Department of Medical Specialties and Public Health, Area of Preventive Medicine and Public Health, Rey Juan Carlos University, Avenida de Atenas S/N, 28922 Madrid, Spain; 2 Department of Medical Specialties and Public Health, Area of Medicine, Rey Juan Carlos University, Avenida de Atenas S/N, 28922 Madrid, Spain

**Keywords:** Lung cancer, Hospitalization, In-hospital mortality, Epidemiology, Spain

## Abstract

**Background:**

Lung cancer is the third most frequent tumor and the main cause of death by tumor in Spain. Although the incidence and mortality are still significantly higher in men than in women, the disparity between the sexes is decreasing. The objective of this study was to analyze the evolution of lung cancer hospitalization and in-hospital mortality rates in Spain from 2010 to 2020.

**Methods:**

The reports of the Minimum Basic Data Set (MBDS) at hospital discharge were used to retrospectively analyze the data of all patients with a primary diagnosis of lung cancer, according to the International Classification of Diseases (ICD-9-CM and ICD-10-CM).

**Results:**

Between 2010 and 2020, there were 315,263 hospitalizations and 70,490 deaths from lung cancer in Spain, the majority (~ 80%) in men. Overall, the rates of hospitalization and in-hospital mortality from lung cancer showed a downward trend throughout the period, although the number of new diagnoses and the absolute number of deaths in women increased. Due to the aging of the population, the degree of comorbidity in patients with lung cancer, although it remains relatively low, is also on the rise.

**Conclusion:**

Lung cancer represents a substantial clinical and economic burden for patients and for the National Health System, so it is necessary to promote primary prevention campaigns, as well as to develop more effective population screening measures to detect cancers early and increase the patient survival.

**Supplementary Information:**

The online version contains supplementary material available at 10.1186/s12885-022-10205-2.

## Background

Lung cancer is the second most common type of cancer and the leading cause of cancer death worldwide [1]. According to estimates from the GLOBOCAN project, in 2020, there were approximately 2.2 million new cases of lung cancer (11.4% of all cancer diagnoses) and 1.8 million deaths associated with the disease worldwide (18.0% of all cancer deaths). In general, lung cancer incidence and mortality rates are approximately two times higher in men than in women, although this relationship varies widely depending on the geographical region [2–4]. In Spain, lung cancer is also one of the main causes of morbidity and mortality, being the third most common tumor in men (13.6%) and in women (6.6%) [5]. In 2020, lung cancer was the leading cause of cancer death in our country, responsible for 21,918 deaths (19.4%). By sex, as occurred worldwide, lung cancer was responsible for the highest number of cancer deaths in men (24.7%), while it was the second leading cause of cancer death in women (11.7%)^1^ [6, 7].

The introduction over the past decade of novel targeted therapies and immune checkpoint inhibitors into the therapeutic armamentarium for patients with stage IV (advanced stage) lung cancer has led to improved survival outcomes, but only in a limited number of patients [8]. The 5-year survival for the majority of patients with late-stage disease is often less than 5%, in contrast to patients diagnosed with early disease (stage IA), who have a > 75% chance of survival over 5 year [9, 10]. Early lung cancer detection improves treatment options, which can result in better survival outcomes. However, due to the late onset of clinical symptoms, lung cancer is frequently diagnosed in advanced stages [11]. Globally, the 5-year net survival from diagnosis for all types of lung cancer combined has been estimated between 10 and 20%. In Spain, from 2000 to 2014, the 5-year net survival increased from 10.8% to 13.5% [12].

Tobacco use is by far the main risk factor for lung cancer, responsible for between 80 and 90% of all cases [13]. The best way to prevent the disease is by avoiding tobacco consumption. Even after a diagnosis of lung cancer, smoking cessation can reduce the risk of progression and death, regardless of the stage of the disease [14]. In this sense, public health measures to prevent the initiation and encourage smoking cessation have contributed to reducing the incidence of lung cancer and improving patient survival, especially in developed countries [15, 16]. In Spain, the prevalence of tobacco consumption has decreased significantly in recent decades, more drastically in men than in women, in part due to the smoking control policies adopted since 2006. In 2020, the estimated prevalence of daily tobacco smoking among people aged 15 and over was 19.8% (23.3% in men and 16.4% in women) [16].

The trend of the incidence of lung cancer generally reflects the temporal variations in the prevalence of tobacco consumption that occurred 20 or 30 years ago [17]. In general, the tobacco epidemic began and reached its peak much earlier in men than in women, so the incidence of lung cancer in men reached its peak and began to decrease at least three decades ago, while in women, it continued to grow in most countries. Although the incidence of the disease is still significantly higher in men than in women, the downward trend in the former and the progressive increase in the latter is reducing the disparity between the sexes [18].

The average age of diagnosis for lung cancer is variable, depending on smoking habits, medical history or sex, among other factors. In general, lung cancer is diagnosed more frequently in individuals between 60 and 75 years of age, being somewhat lower in women than in men [17, 19–22]. According to data from the Spanish Lung Cancer Group’s registry of thoracic tumors, the average age at diagnosis of patients with lung cancer in Spain is 64 years [23]. In the past 50 years, the average age of patients has been increasing progressively [24] along with associated comorbidities [25–27].

Knowledge and monitoring of trends in the incidence and mortality of lung cancer are essential to predict changes in epidemiological patterns that help design and evaluate public health interventions. The objective of this study was to analyze the evolution of hospitalization and in-hospital mortality rates for lung cancer in Spain in the period 2010–2020, the demographic and clinical characteristics of lung cancer patients and the economic impact of the disease to the public health system.

## Materials and methods

### Study design and data source

This is a retrospective, descriptive and observational study with the objective of analyzing the evolution of hospitalization rates and in-hospital mortality due to lung cancer in Spain in the period 2010–2020, as well as to determine differences in the sex and age of patients, and the autonomous community, the health costs associated with the disease and the associated comorbidity index. As a data source, the discharge reports included in the Minimum Basic Data Set (MBDS) were used. The MBDS is a set of basic information on each care episode of each patient that is collected at hospital discharge and consists of both health-related and administrative-related information. The MBDS at hospital discharge has been mandatory by law in Spain since 1987. The MBDS contains 19 variables, of which the 6 most important are: age, sex, main diagnosis, secondary diagnoses, procedures and discharge circumstances [28, 29]. The information collected in the MBDS is coded according to the International Classification of Diseases (ICD-9-CM and ICD-10-CM). All reports of patients with primary diagnosis at discharge of "malignant neoplasia of the bronchi and lung" and "carcinoma in situ of the bronchus and lung" recorded during the period 2010–2020 were included (Table S1).

### Variables and definitions

From each report, the following variables were collected: age (years), sex, destination after discharge (home, transfer or death), duration of hospital stay (days), direct cost of hospital stay (euros), in-hospital mortality and comorbidities at discharge. Comorbidities were weighted according to the Charlson comorbidity index (CCI). This index provides a cumulative evaluation of the patient's condition as one of three levels (level 0, absence of disease; level 1–2, mild or moderate course of the disease; level 3, severe disease) and allows estimating the relative risk of mortality to one year [30].

### Data analysis

The age data were added in six intervals: 39 years or less, 40–49 years, 50–59 years, 60–69 years, 70–79 years and 80 or more years. Both the number of hospitalizations, analyzed by year, sex, age group and autonomous community, as well as the number of deaths, analyzed by year, sex and age group, were described with absolute frequency (n) and/or percentage. The rates of hospitalization and in-hospital mortality, analyzed by year, sex and age group, were expressed per 100,000 population and their confidence intervals. To calculate the rates, the population provided by the National Institute of Statistics, disaggregated by year, sex and age group, was used as the denominator [31], assuming that the age distribution of the population recorded in the MBDS was equivalent to that of the general Spanish population. The length of hospital stay, analyzed by year, was described using the mean and standard deviation. The direct cost per hospital stay, analyzed per year, was estimated from the reference costs of *the Diagnosis Related Groups (DRG)* of the National Health System [32]. Poisson regression models were used to evaluate the differences in the rates of hospitalization and in-hospital mortality, the demographic and clinical characteristics of the patients and the duration and cost of stay between the years of study. Logistic regression analyses were performed to test the association between both age and CCI with the risk of in-hospital mortality. A *p value* < 0.05 was considered statistically significant, and all intervals were calculated with 95% confidence. SPSS Statistics 22.0 software (IBM Corp., New York, USA) was used for statistical analyses. All the data contained in the MBDS records are anonymized; therefore, according to current Spanish legislation, the study did not require formal approval by the Research Ethics Committee of Rey Juan Carlos University.

## Results

From 2010 to 2020, a total of 315,263 patients with lung cancer were hospitalized in Spain, including 247,958 men (78.7%) and 67,305 women (21.3%) (ratio, 3.7). Most patients (99.7%) had a diagnosis of malignant neoplasm of bronchus and lung. The mean age of the patients was 67.3 ± 11.0 years, 68.0 ± 10.5 in men and 64.7 ± 12.2 in women, observing an upward linear trend throughout the study period, from 66.8 ± 11.3 years in 2010 to 68.0 ± 10.5 years in 2020 (*p* < 0.001). The number of hospitalizations decreased by 16.0% in men throughout the study period, while it increased by 61.9% in women (*p* < 0.001 for both comparisons). By age group, the greatest percentage decrease in men was recorded in patients aged 40 to 49 (﻿‒53.1%; *p* < 0.001), and the greatest percentage increase was recorded in women 60 to 69 (117.0%; *p* < 0.001). In women younger than 50 years, however, a significant decrease in the number of hospitalizations was observed, ‒40.2% in women under age 40 (*p* < 0.006) and ‒26.6% in women aged 40–49 (*p* < 0.001) (Table S2).

**Fig. 1:**
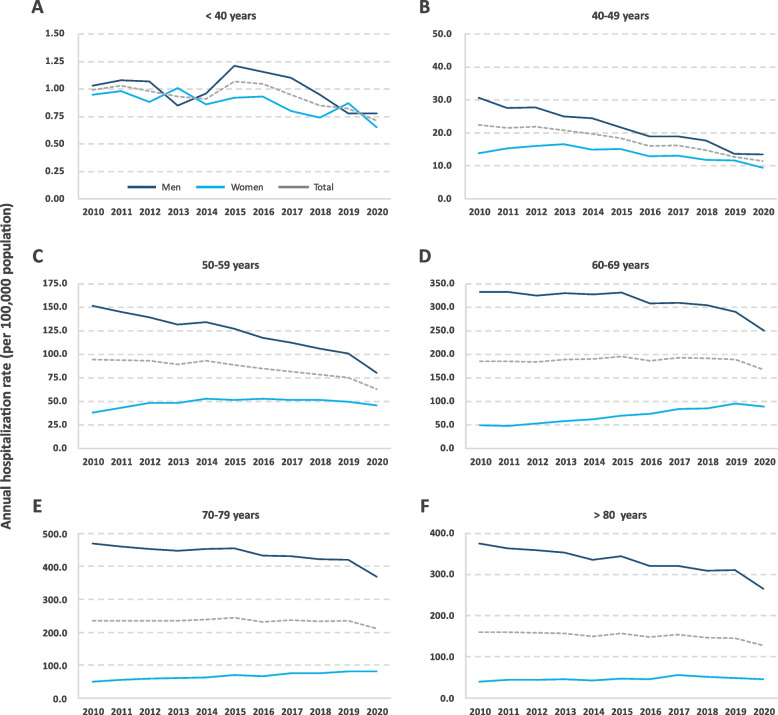
Annual hospitalization rate for lung cancer in men and women in Spain by age group and by year between 2010 and 2020

The annual hospitalization rate (per 100,000 population) during the study period was estimated at 61.38 for both sexes, 98.20 in men and 25.76 in women (ratio, 3.8). By age group, the highest hospitalization rates were recorded in men aged 70–79 years and in women aged 60–69 years, 436.16 and 70.44 per 100,000 population, respectively. In men, a downward trend in the hospitalization rate was observed, with an overall decrease of 16.7%. This decrease was observed in all age groups analyzed. In women, the hospitalization rate showed an upward linear trend throughout the period, recording an increase of 57.9%. By age group, the greatest increase in the rate of hospitalization was observed in women 60 to 69 years. Among women under 50 years of age, however, although the rates remained relatively low throughout the study period compared to those of women 50 years of age and older, the hospitalization rate showed a downward linear trend, recording a decrease of approximately 30% (Table 1 and Fig. 1). Due to the progressive decrease in the hospitalization rates in men and the corresponding increase in women, the male-to-female hospitalization rate ratio gradually decreased throughout the study period, from 5.4 in 2010 to 2.8 in 2020 (‒47.3%).

**Table 1:** Annual hospitalization rate for lung cancer by age group, sex and year in Spain from 2010 to 2020^a^

		**2010**	**2011**	**2012**	**2013**	**2014**	**2015**	**2016**	**2017**	**2018**	**2019**	**2020**	**Total**	***p***
** < 40 years**	Male	1.03	1.08	1.07	0.85	0.96	1.21	1.16	1.10	0.95	0.78	0.78	1.00	0.038
	Female	0.95	0.98	0.88	1.01	0.86	0.92	0.93	0.80	0.74	0.87	0.65	0.88	0.006
	Total	0.99	1.03	0.98	0.93	0.91	1.07	1.05	0.95	0.85	0.82	0.71	0.94	0.001
**40–49 years**	Male	30.68	27.61	27.71	24.98	24.38	21.77	19.01	19.01	17.68	13.67	13.53	21.72	< 0.001
	Female	13.81	15.30	16.05	16.54	14.86	15.04	12.91	13.18	11.91	11.68	9.39	13.66	< 0.001
	Total	22.37	21.55	21.97	20.82	19.69	18.45	16.00	16.13	14.83	12.68	11.47	17.74	< 0.001
**50–59 years**	Male	151.63	145.20	139.21	131.40	134.29	127.04	117.43	112.09	106.02	100.77	80.09	120.95	< 0.001
	Female	38.26	42.82	48.30	48.38	52.56	51.41	52.85	51.25	51.44	49.48	45.96	48.59	< 0.001
	Total	94.43	93.57	93.35	89.51	93.06	88.90	84.87	81.42	78.50	74.91	62.88	84.46	< 0.001
**60–69 years**	Male	332.99	332.63	324.14	330.20	327.00	331.34	308.35	309.99	304.86	290.13	250.58	311.76	< 0.001
	Female	48.77	48.07	53.13	58.07	62.40	69.88	72.86	83.60	84.91	95.53	88.59	70.44	< 0.001
	Total	185.22	184.80	183.38	188.92	189.67	195.65	186.18	192.56	190.77	189.23	166.59	186.52	0.004
**70–79 years**	Male	469.91	460.35	452.62	448.31	453.76	454.87	433.02	430.68	422.05	419.25	368.16	436.16	< 0.001
	Female	49.74	54.48	59.50	61.32	63.24	69.65	65.52	75.10	76.26	81.28	80.66	67.32	< 0.001
	Total	235.73	234.88	234.77	234.55	238.69	243.20	231.56	236.16	233.17	234.81	211.24	233.27	< 0.001
** ≥ 80 years**	Male	375.87	364.04	359.81	353.66	336.47	344.64	321.62	321.26	310.23	310.81	265.39	330.92	< 0.001
	Female	39.68	44.76	44.44	45.28	43.11	47.49	46.36	56.29	51.10	48.41	46.08	46.83	< 0.001
	Total	159.65	159.37	158.42	157.46	150.43	156.78	148.05	154.44	147.17	145.66	127.52	150.94	< 0.001
**Total**	Male	100.66	99.89	99.28	99.97	101.80	103.28	98.27	99.15	97.91	96.14	83.82	98.18	< 0.001
	Female	18.74	20.43	22.37	23.61	24.69	26.62	26.75	29.63	29.66	31.22	29.59	25.77	< 0.001
	Total	59.22	59.66	60.29	61.19	62.60	64.28	61.86	63.74	63.12	63.04	56.17	61.37	0.005

^a^Annual hospitalization rate expressed per 100,000 population

Approximately two-thirds of the patients, 66.7% of men and 65.7% of women, had mild or moderate comorbidities (CCI score 1–2). However, among patients with high comorbidity (CCI score ≥ 3), there were much more men than women, 14.1% versus 6.0%. During the study period, an increasing linear trend was observed in the percentage of patients with high comorbidity, recording a significant increase of 53.6% in 2020 (*p* < 0.001) (Table 2). By autonomous community, the highest rate of hospitalization was recorded in the Principality of Asturias, 92.74 per 100,000 population. The relative risk index (RRI) of hospitalization for lung cancer in this autonomous community, with respect to the national average, was estimated at 1.52 (Table 3).

**Table 2:** Characteristics of patients hospitalized for lung cancer by year in Spain from 2010 to 2020

	**2010**	**2011**	**2012**	**2013**	**2014**	**2015**	**2016**	**2017**	**2018**	**2019**	**2020**	**Total**	***p***
Age (years)^a^	66.8	66.9	66.9	67.1	67.1	67.3	67.3	67.6	67.6	67.9	68.0	67.3	< 0.001
	(± 11.3)	(± 11.3)	(± 11.3)	(± 11.2)	(± 11.1)	(± 11.0)	(± 11.0)	(± 10.9)	(± 10.7)	(± 10.5)	(± 10.5)	(± 11.0)	
Charlson 0^b^	6148	6156	6223	5716	5985	6280	6744	6577	6202	5777	4851	66,659	< 0.001
	(22.3%)	(22.1%)	(22.1%)	(20.0%)	(20.6%)	(21.1%)	(23.5%)	(22.2%)	(21.0%)	(19.5%)	(18.2%)	(21.1%)	
Charlson 1–2	18,609	18,764	18,967	19,456	19,497	19,993	18,650	19,386	19,301	19,397	17,572	209,592	< 0.001
	(67.5%)	(67.3%)	(67.3%)	(68.2%)	(67.0%)	(67.0%)	(64.9%)	(65.4%)	(65.4%)	(65.3%)	(66.1%)	(66.5%)	
Charlson ≥ 3	2818	2962	3004	3340	3600	3560	3342	3696	3992	4523	4175	39,012	< 0.001
	(10.2%)	(10.6%)	(10.7%)	(11.7%)	(12.4%)	(11.9%)	(11.6%)	(12.5%)	(13.5%)	(15.2%)	(15.7%)	(12.4%)	

^a^Age expressed as mean (± SD); ^b^Charlson index expressed as number of patients (%)

**Table 3:** Number of hospitalizations for lung cancer, mean annual hospitalization rate and relative risk index by autonomous community in Spain from 2010 to 2020

Autonomous community	n	%	Annual rate ^a^	IRR	*p*
Principality of Asturias	10,732	3.40	92.74	1.52	< 0.001
Valencian Community	42,197	13.38	76.19	1.25	< 0.001
Castile and León	20,570	6.52	75.55	1.24	< 0.001
Extremadura	8992	2.85	74.97	1.23	< 0.001
Aragon	10,947	3.47	74.94	1.23	< 0.001
Galicia	22,308	7.08	73.99	1.21	< 0.001
Foral Community of Navarra	5224	1.66	73.62	1.21	< 0.001
Basque Country	15,978	5.07	66.2	1.08	< 0.001
Cantabria	3882	1.23	60.17	0.99	0.364
La Rioja	2095	0.66	59.68	0.98	0.296
Catalonia	49,595	15.73	59.51	0.97	< 0.001
Community of Madrid	39,234	12.44	54.62	0.89	< 0.001
Castilla-La Mancha	12,311	3.90	54.11	0.89	< 0.001
Region of Murcia	8654	2.75	53.32	0.87	< 0.001
Canary Islands	11,929	3.78	51.08	0.84	< 0.001
Andalusia	44,430	14.09	48.02	0.79	< 0.001
Balearic Islands	5505	1.75	44.64	0.73	< 0.001
Ceuta	405	0.13	43.83	0.72	< 0.001
Melilla	275	0.09	29.85	0.49	< 0.001

^a^Mean annual hospitalization rate expressed per 100,000 population

IRR, relative risk index

During the period analyzed, a total of 70,490 patients died from lung cancer in Spanish hospitals, of whom 80.9% (*n* = 57,050) were men (ratio, 4.2). The total number of deaths decreased by 15.0% from 2010 to 2020. By sex, the number of deaths decreased by 22.8% in men, while it increased by 29.3% in women (Table S3). The annual in-hospital mortality rate during the study period was estimated at 22.36% for both sexes, 23.01% in men and 19.97% in women (ratio, 1.2). By age group, the highest in-hospital mortality rate, by far, was recorded in patients 80 years of age or older. The risk of in-hospital mortality in this age group was estimated to be almost twofold higher than in patients younger than 40 years (odds ratio [OR] 1.93, 95% CI 1.72–2.17; *p* < 0.001). Likewise, the risk of in-hospital mortality was significantly higher in patients with comorbidities, i.e., more than 2 times higher in patients with a CCI score 1–2 (OR 2.22, 95% CI 2.17–2.28; *p* < 0.001) and almost 3 times higher in patients with a CCI score ≥ 3 (OR 2.88, 95% CI 2.79–2.97; *p* < 0.001).

**Fig. 2:**
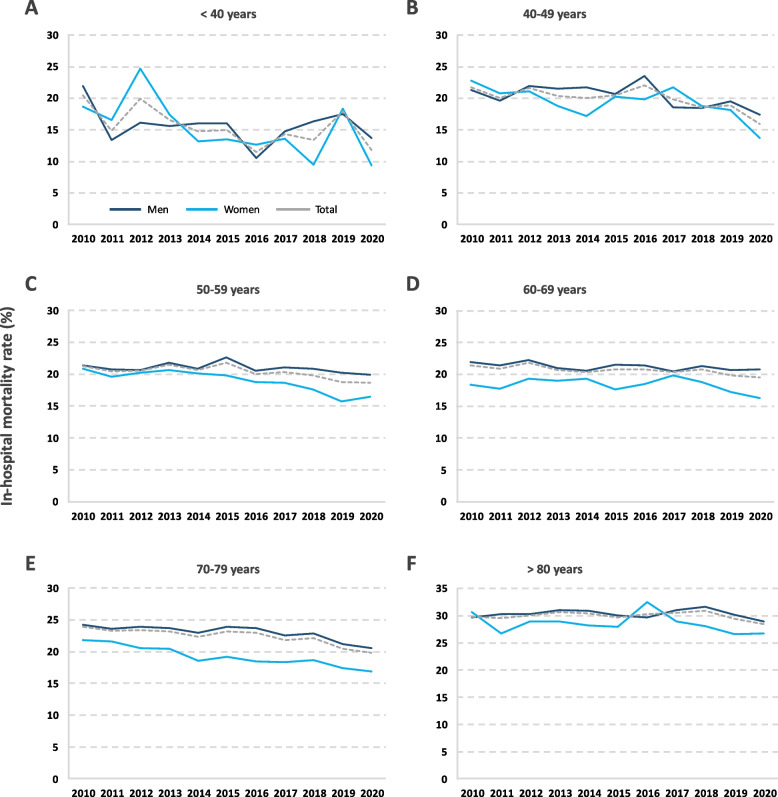
Annual in-hospital mortality rate from lung cancer in men and women in Spain by age group and by year between 2010 and 2020

A decreasing trend was observed in the in-hospital mortality rate during the study period, recording a decrease of 1.91 percentage points in men and 4.40 points in women. However, this decrease was only statistically significant in certain age groups (Table 4 and Fig. 2).

**Table 4:** Annual in-hospital mortality rate from lung cancer by age group, sex and year in Spain from 2010 to 2020^a^

		**2010**	**2011**	**2012**	**2013**	**2014**	**2015**	**2016**	**2017**	**2018**	**2019**	**2020**	**Total**	***p***
** < 40 years**	Male	21 .95	13 .39	16 .13	15 .63	16 .04	16 .03	10 .57	14 .78	16 .33	17 .50	13 .75	15 .63	0.373
	Female	18 .69	16 .51	24 .74	17 .43	13 .19	13 .54	12 .63	13 .58	9 .46	18 .39	9 .38	15 .64	0.044
	Total	20 .43	14 .83	19 .91	16 .59	14 .72	14 .98	11 .47	14 .29	13 .37	17 .96	11 .81	15 .63	0.043
**40–49 years**	Male	21 .30	19 .65	21 .92	21 .47	21 .71	20 .71	23 .55	18 .57	18 .44	19 .48	17 .45	20 .61	0.084
	Female	22 .74	20 .79	21 .11	18 .73	17 .15	20 .21	19 .88	21 .71	18 .82	18 .14	13 .70	19 .53	0.022
	Total	21 .74	20 .05	21 .63	20 .40	20 .01	20 .51	22 .09	19 .84	18 .59	18 .87	15 .92	20 .20	0.004
**50–59 years**	Male	21 .43	20 .70	20 .61	21 .83	20 .87	22 .66	20 .52	21 .08	20 .88	20 .21	19 .95	21 .02	0.252
	Female	20 .85	19 .61	20 .25	20 .67	20 .12	19 .82	18 .81	18 .62	17 .56	15 .75	16 .51	18 .83	< 0.001
	Total	21 .31	20 .45	20 .52	21 .51	20 .66	21 .84	19 .98	20 .30	19 .78	18 .72	18 .68	20 .38	< 0.001
**60–69 years**	Male	21 .92	21 .38	22 .29	21 .04	20 .59	21 .51	21 .36	20 .46	21 .34	20 .72	20 .74	21 .21	0.040
	Female	18 .36	17 .75	19 .29	18 .96	19 .26	17 .65	18 .45	19 .86	18 .82	17 .24	16 .29	18 .26	0.105
	Total	21 .43	20 .89	21 .84	20 .70	20 .36	20 .79	20 .77	20 .32	20 .76	19 .81	19 .51	20 .63	0.001
**70–79 years**	Male	24 .16	23 .52	23 .83	23 .67	22 .93	23 .86	23 .72	22 .54	22 .87	21 .17	20 .57	22 .99	< 0.001
	Female	21 .80	21 .59	20 .56	20 .38	18 .56	19 .19	18 .47	18 .33	18 .69	17 .40	16 .81	18 .99	< 0.001
	Total	23 .88	23 .27	23 .37	23 .19	22 .29	23 .13	22 .91	21 .81	22 .12	20 .45	19 .78	22 .36	< 0.001
** ≥ 80 years**	Male	29 .64	30 .23	30 .32	30 .97	30 .86	30 .05	29 .67	30 .98	31 .65	30 .19	28 .93	30 .34	0.995
	Female	30 .68	26 .70	28 .91	28 .97	28 .16	27 .97	32 .48	28 .94	28 .03	26 .64	26 .72	28 .52	0.369
	Total	29 .80	29 .59	30 .07	30 .61	30 .37	29 .65	30 .23	30 .51	30 .86	29 .44	28 .43	29 .98	0.599
**Total**	Male	23 .54	23 .01	23 .54	23 .41	22 .84	23 .64	23 .21	22 .71	23 .16	22 .15	21 .63	23 .01	< 0.001
	Female	21 .88	20 .68	21 .42	21 .00	20 .19	20 .08	20 .41	20 .60	19 .58	18 .09	17 .48	19 .97	< 0.001
	Total	23 .27	22 .60	23 .14	22 .94	22 .31	22 .89	22 .59	22 .21	22 .31	21 .13	20 .52	22 .36	< 0.001

^a^Annual in-hospital mortality rate expressed as percentage of deaths per inpatient population

The mean hospital stay throughout the study period was 9.54 ± 9.45 days, with a clear downward trend in the duration of admissions, from 10.42 ± 9.67 days in 2010 to 9.08. ± 8.84 days in 2020 (﻿‒12.9%; *p* < 0.001); from 2014 to 2017, however, the length of stay remained more or less stable. The average direct cost per hospitalized patient during the study period was estimated at 5,366.4 ± 4,508.4 EUR, being similar in men and women. The minimum average cost was 5,110.2 ± 3,271.1 EUR in 2017, and the maximum was 5,574.4 ± 6,299.6 EUR in 2011 (Table 5).

**Table 5:** Length of hospital stay and direct cost per hospital stay for lung cancer by year in Spain from 2010 to 2020

	**2010**	**2011**	**2012**	**2013**	**2014**	**2015**	**2016**	**2017**	**2018**	**2019**	**2020**	**Total**	***p***
Length of stay^a^	10.4	10.0	9.7	9.5	9.4	9.5	9.5	9.5	9.3	9.2	9.1	9.5	< 0.001
	(± 9.7)	(± 9.6)	(± 9.2)	(± 8.7)	(± 9.2)	(± 8.8)	(± 9.0)	(± 9.7)	(± 9.6)	(± 9.1)	(± 8.8)	(± 9.4)	
Direct cost	5479	5574	5407	5560	5171	5522	5118	5110	5178	5393	5554	5366	< 0.001
	(± 6515)	(± 6300)	(± 6028)	(± 4911)	(± 3738)	(± 3449)	(± 3212)	(± 3271)	(± 3390)	(± 3311)	(± 3725)	(± 4508)	

^a^Length of stay (days) and direct cost (euros) expressed as mean (± SD)

## Discussion

According to the data collected in this retrospective nationwide study from 2010 to 2020, there were more than 300,000 hospitalizations and approximately 70,500 deaths from lung cancer in Spain, which implies a substantial clinical and economic burden for both patients and the National Health System [6, 7]. The annual rate of hospitalization was much higher in men than in women, with a male-to-female ratio of 3.8. The sex disparity in the incidence of different types of cancer, particularly lung cancer, has been widely reported in numerous epidemiological studies [33–35]. According to data from the GLOBOCAN project, the incidence of lung cancer is approximately two times higher in men than in women globally, although the male-to-female ratio varies depending on the geographical area, from 1.2 in North America to 5.6 in Northern African countries [2]. During the study period, we observed a downward trend in the rate of hospitalization in men and an upward trend in women (‒16.7% versus + 60%, respectively), reducing the male-to-female ratio from 5.4 in 2010 to 2.8 in 2020, a proportion that was very similar to the average found in countries of southern Europe in the same year, estimated at 2.6 [2, 18]. This sex disparity, both in hospitalization rates and in temporal evolution, has already been observed in previous studies. During the period 2001–2011, a significant downward trend was observed in hospitalization rates in men, registering a slight decrease of 4.3%, from 112.5 to 107.7 per 100,000 population, while in women, the trend was ascending, registering a strong increase of 100.3%, from 11.8 to 23.6 per 100,000 population (*p* < 0.001 for both sexes). Consequently, the proportion between sexes was reduced to less than half throughout the decade, from 9.6 in 2001 to 4.6 in 2011 (‒52.2%) [36]. Considering the data from both studies, in the past 20 years, the rate of hospitalization for lung cancer in Spain has decreased by approximately 25% in men, while it has increased by more than 150% in women, reducing by more than 70% the difference between the sexes (9.6 in 2001 compared to 2.8 in 2020). In a recently published population study, which included incidence data from 1978 to 2012 in 45 countries of the five continents, a downward trend was observed in men in 19 countries, and an upward trend was observed in women in 26 countries. Sex differences were observed in most countries, especially in Central and Eastern European countries. Likewise, the gap between men and women has been narrowing globally over the past three decades, particularly in Northern Europe, Eastern Europe, North America and Oceania [18]. This study highlights the upward trend in the incidence of lung cancer in men in Spain until approximately 1990 —a trend that had been recorded since at least the 1950s [15] —and its subsequent stabilization for approximately a decade until the beginning of the downward trend observed in the past 20 years. According to these two studies, the incidence in women in Spain remained low and stable until the beginning of the 1990s, after which it began to increase steadily [15, 18].

Although the differences in the incidence of lung cancer between men and women can be attributed to different factors, tobacco consumption is the main cause of the disease, and variations in smoking patterns are closely associated with this disparity, particularly in Western countries [35]. The incidence and mortality from lung cancer increase in the two or three decades after the peak of tobacco consumption [17]. In industrialized countries, this peak was reached earlier in men than in women, which explains the historically higher incidence rates in men [37, 38]. In Spain, the maximum prevalence of tobacco consumption in men was reached in the mid-1970s (﻿birth cohort of 1950–1959) and, after stabilizing for approximately a decade, began to decrease. In women, the maximum prevalence was reached approximately two decades later, in the 1990s (birth cohort of 1960–1969), and its effects are currently reflected in the upward trend in the incidence of lung cancer among women [39].

With the implementation of regulations aimed at restricting tobacco consumption in Spain (Law 28/2005 and Law 42/2010), the prevalence of habitual tobacco consumption from 2006 to 2020 has decreased substantially, by 26.3% in men and 23.7% in women. However, the percentage of smokers is still high, 19.8% among the population aged 15 and older, and it remains higher in men than in women, 23.3% compared to 16.4% (ratio, 1. 4) [16, 40]. The average prevalence in the 27 member countries of the EU is similar to the Spanish prevalence, 18.4% (22.3% in men and 14.8% in women), although the variation between countries is high [41].

By age group, the highest annual hospitalization rate for lung cancer was recorded in men aged 70 to 79 years (436.16 per 100,000 population) and in women aged 60 to 69 years (70.44 per 100,000 population), although in the latter case, the hospitalization rate was very similar to that recorded in the higher age group, 70–79 years (67.32 per 100,000 population). Due to the time delay between the onset of smoking and the manifestation of lung cancer, the disease is exceedingly rare in people under 40 years of age (< 0.1% in this study). On the other hand, although the hospitalization rates increase with age, they generally begin to decrease in people older than 80, probably due to the presence of a competitive risk of mortality from other causes or due to an incomplete diagnosis [42].

Similarly, during the previous decade, 2001–2011, the age group with the highest incidence of lung cancer in Spain was also that of the 70 to 79 age group, in both men (492.78 per 100,000 population) and women (45.5 per 100,000 population) [36]. While the distribution of the relative incidence in the different age groups has remained more or less stable in men in the past two decades (2001–2020), in women, the higher relative incidence has been weighted toward younger individuals in the last decades. According to the last Annual Report of the Spanish National Health System, during the period 1993–2020, the prevalence of daily tobacco consumption among the youngest men, ages 15 to 24, remained below the prevalence for total male consumption every year, while consumption in younger women was much higher than that of the total female population until 2014, the year in which the percentages began to decrease. In 2001, consumption among women aged 15 to 24 years almost equaled total male consumption (36.9% compared to 39.2%) [16]. The displacement of the peak incidence of the disease to younger ages in women reflects the relative increase in tobacco consumption among adolescents in recent decades [43–45]. The mean age of hospitalization in this study was estimated at 68.0 in men and 64.7 in women, with a clear upward trend during the period. This increase in the average age of the patients probably reflects the increase in hospitalization rates in men aged 70 or older, almost 5%, with greater specific weight than the relative decrease in the age of hospitalization in women.

The majority of patients in this study, approximately 65%, had mild or moderate comorbidity (CCI score 1–2), although the percentage of patients with high comorbidity (CCI score ≥ 3) increased progressively throughout the study period, an increase that was already observed in the previous decade [36]. It is known that the prevalence of comorbidities increases with age, making cancer patients more vulnerable [25–27]. The percentage of male patients aged 80 years or older increased by 8% throughout the study period, while in women, this percentage decreased by 13.4%. The implementation of strategies for the clinical management of comorbidities is essential to optimize treatments and improve the survival of older adults with lung cancer.

Between 2010 and 2020, more than 70,000 patients died from lung cancer in Spanish hospitals, 80% of whom were men. The net number of annual in-hospital deaths from lung cancer decreased over the decade by 22.8% in men, while it increased by 29.3% in women, thus reducing the male-to-female ratio of in-hospital mortality from 5.6 in 2010 to 3.4 in 2020. In-hospital mortality rates, however, were very similar between men and women throughout the study period, with a much higher decrease observed in women than in men, 20.1% versus 8.1%. In both sexes, the highest in-hospital mortality rate was recorded in patients aged 80 or older, although the presence of competing risks of death from other causes is an important factor to take into account [42]. In all other age groups, the rates were very similar and slightly higher in men than in women (21.5% versus 18.9%). According to our results, older age and the presence of comorbidities are significantly associated with an increased risk of in-hospital mortality. Previous studies have shown that there are differences associated with sex in the clinicopathological characteristics and survival of patients with lung cancer, such that women have a lower risk of progression and death than men [46, 47]. According to a report prepared by REDECAN, during the period 2008–2013, the net survival at 5 years post-diagnosis in patients with lung cancer (all stages) was 12% in men and 18% in women. Net survival in Spain has improved in recent decades, similar to that of neighboring countries. In men, it went from 11.2% in the 2002–2007 period to 12.7% in the 2008–2013 period (+ 13.4%), while in women, it went from 16.2% to 17.6% in the same period of time (+ 8.6%) [48]. In a recent study carried out by Spanish researchers, in which data from patients with advanced non-small-cell lung cancer were analyzed, the median overall survival was estimated at 12 months for men and 19 months for women (HR 0.77; 95% CI, 0.68–0.87; *p* < 0.001) [49]. Despite these advances, the mortality rate continues to increase in women due to the progressive increase in the incidence of the disease. In Spain, lung cancer mortality rates have shown a downward trend in men and an upward trend in women since the early 1990s [50]. These patterns are similar to those observed in other European countries [51, 52]. Although in Spain mortality in men is still much higher than in women, the proportion between sexes has drastically declined in the past three decades, from 13.3 in 1989 to 3.7 in 2018 [50]. In Europe, the corresponding proportion decreased from 5.1 in 1994 to 2.2 in 2019 [53].

One of the main reasons for the poor prognosis of lung cancer is that, due to the late onset of symptoms, up to 70% of patients present with advanced stage disease (stage III or IV) at diagnosis and thus the therapeutic options for curative treatment at this time are limited [9, 10, 54]. To date, the main strategy shown to substantially reduce lung cancer mortality is based on early detection in asymptomatic individuals [55]. Several randomized clinical trials conducted in the United States and Europe have demonstrated the benefit of low-dose computed tomography (LDCT) thorax imaging for the secondary prevention of lung cancer [56, 57]. The National Lung Screening Trial (NLST) and the Nederlands-Leuvens Longkanker Screenings Onderzoek (NELSON) trial are two trials powered to evaluate reduction in lung cancer mortality. These studies have provided conclusive evidence of a mortality reduction associated with LDCT screening in high-risk populations. The NLST reported a 20% (95% CI, 6.8–26.7; *p* = 0.004) overall reduction in lung cancer mortality after 6.5-years follow-up when comparing LDCT to chest X-ray for lung cancer screening [58, 59]. More recently, the NELSON trial published their final results, reporting a cumulative rate ratio for death from lung cancer at 10 years of follow-up of 0.76 (95% CI, 0.61–0.94; *p* = 0.01) when comparing LDCT to no screening [60]. Other randomized trials conducted in Europe, although not powered to evaluate reduction in lung cancer mortality, have reported similar encouraging results [56, 57]. Nonetheless, there are important issues for the successful global implementation of LDCT lung cancer screening programs that remain to be resolved, among the most important, the optimal selection of the screening population, the screening interval, the appropriate nodule management protocol, the adherence of participants, the cost-effectiveness or the necessary infrastructure [61].

During the study period, we observed a decrease in the average hospital stay, which was reduced by almost 13%. This decrease was particularly pronounced in the first four years of the period. This downward trend in the duration of hospital stay had been observed in the previous decade, in which there was a somewhat greater decline of 20.5% [36]. This decrease in the length of stay was not accompanied by a significant variation in the average direct cost per hospitalized patient, contrary to what was observed in the previous decade, in which costs increased by approximately 20% [36]. In general, it is difficult to establish comparisons with respect to the direct costs of lung cancer in other countries, since the variables analyzed in other studies are not well defined or are not comparable. On the other hand, the average direct cost of the hospital stay managed in this study is an approximation obtained from the reference costs of the DRGs of the National Health System. In any case, the global analysis of the trends in health costs derived from lung cancer allows us to conclude that the diagnostic delay prompts a substantial increase in health expenditures that could be avoided with primary prevention measures and early diagnostic screening [62].

This study has some limitations. The number of new diagnoses of lung cancer has been estimated from the number of cases included in the main diagnostic variable of the MBDS, but we do not know the number of cases coded as a secondary diagnosis that, if significant, could result in an underestimation of the new cases. In any case, this does not prevent us from analyzing the general trend in terms of the evolution of new lung cancer diagnoses. Regarding mortality rates, only deaths that occurred in the hospital setting were analyzed, without taking into account those that occurred outside this context, for example, terminal patients undergoing palliative home treatment. The new database of the MBDS eliminates the variable “readmission”, so we cannot know the real percentage of cases that have been counted as new diagnoses, which may be readmissions. Some variables of interest, such as smoking, tumor histology or the clinical stage of cancer, are not coded in the database, so they could not be considered in the study. Finally, statements related to causality cannot be made in this study as it is purely an observational study.

## Conclusion

In summary, lung cancer represents a substantial clinical and economic burden for both patients and the National Health System. Between 2010 and 2020, a downward trend in the number of hospital admissions for lung cancer was observed in Spain, due in part to the implementation of anti-smoking policies, which have efficiently contributed to the reduction of the smoking prevalence. However, the number of hospital admissions among certain age groups of women showed an upward trend, most likely due to their later incorporation to the smoking habit. Although the incidence is still much higher in men than in women, the disparity between the sexes has been narrowing in recent decades. The degree of comorbidity in patients with lung cancer remains relatively low, but it is increasing due to the aging of the population, which can have a negative impact on treatment and survival. The mortality rate due to lung cancer is decreasing globally. However, despite the diagnoses of cancer in women at earlier ages than men, the absolute number of deaths in women continues to rise. Mortality from lung cancer is still much higher in men than in women, but the sex ratio has been drastically reduced in the past three decades. We must continue joining efforts to find more effective population screening measures that allow for early diagnosis and increase patient survival, without forgetting the importance of primary prevention campaigns.

## Supplementary Information

**Additional file 1: Table S1.** Definition of neoplasms of the respiratory system used according to the ICD-9-CM and ICD-10-CM codes.

**Additional file 2: Table S2.** Number of hospitalizations for lung cancer by age group, sex and year in Spain from 2010 to 2020.

**Additional file 3: Table S3. **Number of in-hospital lung cancer deaths by age group, sex and year in Spain from 2010 to 2020.

## Data Availability

The dataset supporting the conclusions of this article is available in the MBDS repository, https://www.mscbs.gob.es/en/estadEstudios/estadisticas/cmbdhome.htm. The information contained in this repository is in the public domain and can be accessed without the need for any administrative permissions.

## Footnotes

1 These estimates do not reflect the impact of the COVID-19 pandemic and may vary slightly from the actual figures.
