# Supplementary material for: Trends in hospitalization and in-hospital mortality rates among patients with lung cancer in Spain between 2010 and 2020
Source: BMC Cancer. 2022 Nov 21;22:1199. doi: 10.1186/s12885-022-10205-2 (PMC9680125; doi:10.1186/s12885-022-10205-2)
Supplement: Supplementary file 1 — Additional file 1: Table S1. Definition of neoplasms of the respiratory system used according to the ICD-9-CM and ICD-10-CM codes. [file 12885_2022_10205_MOESM1_ESM.docx]

| **Table S1. Definition of neoplasms of the respiratory system used according to the ICD-9-CM and ICD-10-CM codes** | |
| --- | --- |
| **ICD‑9‑CM (cases collected from 2010 to 2015)** | |
| *Malignant neoplasm of trachea, bronchus and lung* | |
| 162.2 | Malignant neoplasm of main bronchus |
| 162.3 | Malignant neoplasm of upper lobe, bronchus or lung |
| 162.4 | Malignant neoplasm of middle lobe, bronchus or lung |
| 162.5 | Malignant neoplasm of lower lobe, bronchus or lung |
| 162.8 | Malignant neoplasm of other parts of bronchus or lung |
| 162.9 | Malignant neoplasm of bronchus and lung, unspecified |
| *Carcinoma in situ of respiratory system* | |
| 231.2 | Carcinoma *in situ* of bronchus and lung |
| **ICD‑10‑CM (cases collected from 2016 to 2020)** | |
| *Malignant neoplasm of bronchus and lung* | |
| C34.00 | Malignant neoplasm of unspecified main bronchus |
| C34.01 | Malignant neoplasm of right main bronchus |
| C34.02 | Malignant neoplasm of left main bronchus |
| C34.10 | Malignant neoplasm of upper lobe, unspecified bronchus or lung |
| C34.11 | Malignant neoplasm of upper lobe, right bronchus or lung |
| C34.12 | Malignant neoplasm of upper lobe, left bronchus or lung |
| C34.2 | Malignant neoplasm of middle lobe, bronchus or lung |
| C34.30 | Malignant neoplasm of lower lobe, unspecified bronchus or lung |
| C34.31 | Malignant neoplasm of lower lobe, right bronchus or lung |
| C34.32 | Malignant neoplasm of lower lobe, left bronchus or lung |
| C34.80 | Malignant neoplasm of overlapping sites of unspecified bronchus and lung |
| C34.81 | Malignant neoplasm of overlapping sites of right bronchus and lung |
| C34.82 | Malignant neoplasm of overlapping sites of left bronchus and lung |
| C34.90 | Malignant neoplasm of unspecified part of unspecified bronchus or lung |
| C34.91 | Malignant neoplasm of unspecified part of right bronchus or lung |
| C34.92 | Malignant neoplasm of unspecified part of left bronchus or lung |
| *Carcinoma in situ of bronchus and lung* | |
| D02.20 | Carcinoma *in situ* of unspecified bronchus and lung |
| D02.21 | Carcinoma *in situ* of right bronchus and lung |
| D02.22 | Carcinoma *in situ* of left bronchus and lung |
