# Supplementary material for: Trends in hospitalization and in-hospital mortality rates among patients with lung cancer in Spain between 2010 and 2020
Source: BMC Cancer. 2022 Nov 21;22:1199. doi: 10.1186/s12885-022-10205-2 (PMC9680125; doi:10.1186/s12885-022-10205-2)
Supplement: Supplementary file 2 — Additional file 2: Table S2. Number of hospitalizations for lung cancer by age group, sex and year in Spain from 2010 to 2020. [file 12885_2022_10205_MOESM2_ESM.docx]

| **Table S2. Number of hospitalizations for lung cancer by age group, sex and year in Spain from 2010 to 2020** | | | | | | | | | | | | | |
| --- | --- | --- | --- | --- | --- | --- | --- | --- | --- | --- | --- | --- | --- |
|  |  | **2010** | **2011** | **2012** | **2013** | **2014** | **2015** | **2016** | **2017** | **2018** | **2019** | **2020** | **Total** |
| **< 40 years** | Male | 123 | 127 | 124 | 96 | 106 | 131 | 123 | 115 | 98 | 80 | 80 | 1203 |
|  | Female | 107 | 109 | 97 | 109 | 91 | 96 | 95 | 81 | 74 | 87 | 64 | 1010 |
|  | Total | 230 | 236 | 221 | 205 | 197 | 227 | 218 | 196 | 172 | 167 | 144 | 2213 |
| **40-49 years** | Male | 1136 | 1038 | 1054 | 955 | 935 | 840 | 739 | 743 | 694 | 539 | 533 | 9206 |
|  | Female | 497 | 558 | 592 | 614 | 554 | 564 | 488 | 502 | 457 | 452 | 365 | 5643 |
|  | Total | 1633 | 1596 | 1646 | 1569 | 1489 | 1404 | 1227 | 1245 | 1151 | 991 | 898 | 14849 |
| **50-59 years** | Male | 4293 | 4232 | 4153 | 4000 | 4192 | 4068 | 3845 | 3743 | 3601 | 3484 | 2812 | 42423 |
|  | Female | 1103 | 1270 | 1467 | 1500 | 1670 | 1675 | 1760 | 1740 | 1777 | 1740 | 1641 | 17343 |
|  | Total | 5396 | 5502 | 5620 | 5500 | 5862 | 5743 | 5605 | 5483 | 5378 | 5224 | 4453 | 59766 |
| **60-69 years** | Male | 7244 | 7354 | 7345 | 7611 | 7615 | 7793 | 7361 | 7552 | 7576 | 7378 | 6548 | 81377 |
|  | Female | 1149 | 1149 | 1301 | 1445 | 1568 | 1773 | 1875 | 2195 | 2274 | 2616 | 2493 | 19838 |
|  | Total | 8393 | 8503 | 8646 | 9056 | 9183 | 9566 | 9236 | 9747 | 9850 | 9994 | 9041 | 101215 |
| **70-79 years** | Male | 7264 | 7140 | 6965 | 6920 | 7095 | 7229 | 6989 | 7071 | 7158 | 7427 | 6666 | 77924 |
|  | Female | 968 | 1056 | 1138 | 1168 | 1212 | 1350 | 1283 | 1489 | 1557 | 1730 | 1755 | 14706 |
|  | Total | 8232 | 8196 | 8103 | 8088 | 8307 | 8579 | 8272 | 8560 | 8715 | 9157 | 8421 | 92630 |
| **≥ 80 years** | Male | 3101 | 3156 | 3249 | 3345 | 3309 | 3488 | 3353 | 3412 | 3305 | 3293 | 2814 | 35825 |
|  | Female | 590 | 693 | 709 | 749 | 735 | 826 | 825 | 1016 | 924 | 871 | 827 | 8765 |
|  | Total | 3691 | 3849 | 3958 | 4094 | 4044 | 4314 | 4178 | 4428 | 4229 | 4164 | 3641 | 44590 |
| **Total** | Male | 23161 | 23047 | 22890 | 22927 | 23252 | 23549 | 22410 | 22636 | 22432 | 22201 | 19453 | 247958 |
|  | Female | 4414 | 4835 | 5304 | 5585 | 5830 | 6284 | 6326 | 7023 | 7063 | 7496 | 7145 | 67305 |
|  | Total | 27575 | 27882 | 28194 | 28512 | 29082 | 29833 | 28736 | 29659 | 29495 | 29697 | 26598 | 315263 |
