# Supplementary material for: Trends in hospitalization and in-hospital mortality rates among patients with lung cancer in Spain between 2010 and 2020
Source: BMC Cancer. 2022 Nov 21;22:1199. doi: 10.1186/s12885-022-10205-2 (PMC9680125; doi:10.1186/s12885-022-10205-2)
Supplement: Supplementary file 3 — Additional file 3: Table S3. Number of in-hospital lung cancer deaths by age group, sex and year in Spain from 2010 to 2020. [file 12885_2022_10205_MOESM3_ESM.docx]

| **Table S3. Number of in-hospital lung cancer deaths by age group, sex and year in Spain from 2010 to 2020** | | | | | | | | | | | | | |
| --- | --- | --- | --- | --- | --- | --- | --- | --- | --- | --- | --- | --- | --- |
|  |  | **2010** | **2011** | **2012** | **2013** | **2014** | **2015** | **2016** | **2017** | **2018** | **2019** | **2020** | **Total** |
| **< 40 years** | Male | 27 | 17 | 20 | 15 | 17 | 21 | 13 | 17 | 16 | 14 | 11 | 188 |
|  | Female | 20 | 18 | 24 | 19 | 12 | 13 | 12 | 11 | 7 | 16 | 6 | 158 |
|  | Total | 47 | 35 | 44 | 34 | 29 | 34 | 25 | 28 | 23 | 30 | 17 | 346 |
| **40-49 years** | Male | 242 | 204 | 231 | 205 | 203 | 174 | 174 | 138 | 128 | 105 | 93 | 1897 |
|  | Female | 113 | 116 | 125 | 115 | 95 | 114 | 97 | 109 | 86 | 82 | 50 | 1102 |
|  | Total | 355 | 320 | 356 | 320 | 298 | 288 | 271 | 247 | 214 | 187 | 143 | 2999 |
| **50-59 years** | Male | 920 | 876 | 856 | 873 | 875 | 922 | 789 | 789 | 752 | 704 | 561 | 8917 |
|  | Female | 230 | 249 | 297 | 310 | 336 | 332 | 331 | 324 | 312 | 274 | 271 | 3266 |
|  | Total | 1150 | 1125 | 1153 | 1183 | 1211 | 1254 | 1120 | 1113 | 1064 | 978 | 832 | 12183 |
| **60-69 years** | Male | 1588 | 1572 | 1637 | 1601 | 1568 | 1676 | 1572 | 1545 | 1617 | 1529 | 1358 | 17263 |
|  | Female | 211 | 204 | 251 | 274 | 302 | 313 | 346 | 436 | 428 | 451 | 406 | 3622 |
|  | Total | 1799 | 1776 | 1888 | 1875 | 1870 | 1989 | 1918 | 1981 | 2045 | 1980 | 1764 | 20885 |
| **70-79 years** | Male | 1755 | 1679 | 1660 | 1638 | 1627 | 1725 | 1658 | 1594 | 1637 | 1572 | 1371 | 17916 |
|  | Female | 211 | 228 | 234 | 238 | 225 | 259 | 237 | 273 | 291 | 301 | 295 | 2792 |
|  | Total | 1966 | 1907 | 1894 | 1876 | 1852 | 1984 | 1895 | 1867 | 1928 | 1873 | 1666 | 20708 |
| **≥ 80 years** | Male | 919 | 954 | 985 | 1036 | 1021 | 1048 | 995 | 1057 | 1046 | 994 | 814 | 10869 |
|  | Female | 181 | 185 | 205 | 217 | 207 | 231 | 268 | 294 | 259 | 232 | 221 | 2500 |
|  | Total | 1100 | 1139 | 1190 | 1253 | 1228 | 1279 | 1263 | 1351 | 1305 | 1226 | 1035 | 13369 |
| **Total** | Male | 5451 | 5302 | 5389 | 5368 | 5311 | 5566 | 5201 | 5140 | 5196 | 4918 | 4208 | 57050 |
|  | Female | 966 | 1000 | 1136 | 1173 | 1177 | 1262 | 1291 | 1447 | 1383 | 1356 | 1249 | 13440 |
|  | Total | 6417 | 6302 | 6525 | 6541 | 6488 | 6828 | 6492 | 6587 | 6579 | 6274 | 5457 | 70490 |
